# Encapsulation efficiency determination methods for the analysis of different antigenic peptides within phosphatidylserine-liposomes

**DOI:** 10.1007/s00216-026-06453-x

**Published:** 2026-03-25

**Authors:** Irene Latorre, Montserrat Mancera-Arteu, Oumaima El Ouahabi, Míriam Salvadó, Sílvia Rodríguez-Vidal, Victoria Sanz-Nebot

**Affiliations:** 1 Department of Chemical Engineering and Analytical Chemistry, Institute for Research on Nutrition and Food Safety (INSA·UB), University of Barcelona, Martí i Franquès 1-11, 08028 Barcelona, Spain; 2 Ahead Therapeutics SL, Barcelona, Spain

**Keywords:** Autoimmune diseases, PS-Liposomes, Encapsulation efficiency, Autoantigenic peptides, Liquid chromatography, Ultracentrifugation

## Abstract

The prevalence of autoimmune diseases (AIDs) is increasing and persists in individuals of all ages as reported by Pisetsky (*Nat Rev Nephrol*, 2023). Only palliative treatments are currently available as the development of a treatment that selectively eliminates the immune system autoreaction, without affecting its normal functioning, is not an easy task. In this regard, an innovative strategy based on phosphatidylserine-liposomes (PS-Liposomes) loaded with the autoantigenic peptide that triggers the autoimmune attack has demonstrated efficacy in different AIDs as described by Almenara-Fuentes et al. (*Nanomedicine* 48, 2023) and Pujol-Autonell et al. (*PLoS One* 10, 2015). Encapsulation efficiency (EE) determination in liposomal therapeutic formulations is considered one of the most important attributes, as the encapsulated drug concentration is usually related to the treatment’s effectiveness. In this regard, several methods have been described in the literature for the determination of specific peptides within liposomes. However, the described methodologies cannot usually be applied to other peptides with different properties. In this study, peptides with different lengths and physicochemical properties encapsulated in PS-Liposome formulations were used as models to evaluate several methods for both free and total antigen quantification. Different approaches for liposome lysis and subsequent total or encapsulated antigen quantification were studied. On the other hand, a comprehensive comparison of different centrifugal filters and an ultracentrifugation method was performed for free antigen determination. Identification and quantification of the autoantigenic peptides were performed by reversed-phase high-performance liquid chromatography with ultraviolet detection (RP-HPLC-UV). Results obtained for direct and indirect EE determinations were compared for PS-Liposome formulations, and the best conditions for each peptide were selected.

**Graphical Abstract:**

**Figure:**
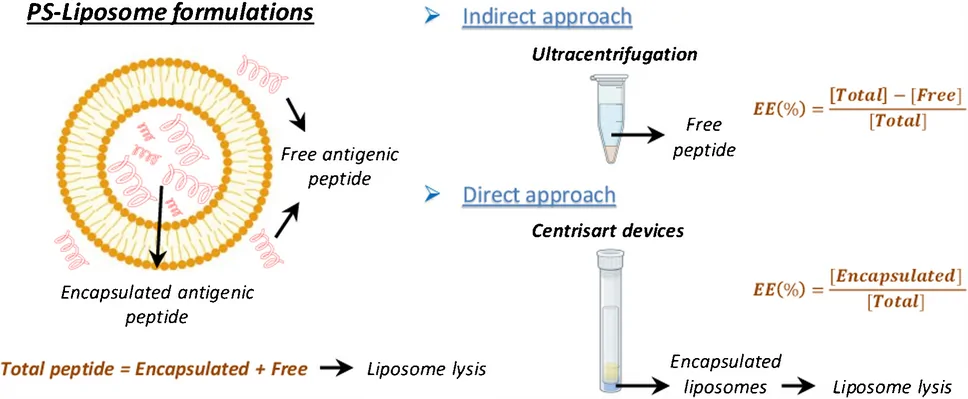

**Supplementary Information:**

The online version contains supplementary material available at https://doi.org/10.1007/s00216-026-06453-x.

## Introduction

Autoimmune diseases (AIDs) are characterised by a malfunction of the immune system resulting in the production of defective T and B cells that attack their own healthy tissues and cells [1]. Current AIDs treatments are based on palliative immunosuppressive tactics by employing agents with a wide range of action, resulting in a lack of disease-specificity [2, 3]. In this regard, a nanotechnology approach that relies on peripherally induced tolerance mechanisms is being developed. Specific antigens are encapsulated in liposomes that present phosphatidylserine in the lipid bilayer (PS-Liposomes). PS-Liposomes mimic apoptotic cell’s structure to replicate the ability of the apoptosis process to induce immunologic tolerance. In this way, specific peripheral tolerance towards the encapsulated antigen is induced in the long term [1, 4]. The effectiveness of this antigen-specific strategy has already been proven in various autoimmunity disorders, such as multiple sclerosis (MS) [4, 5] and type 1 diabetes (T1D) [6], among others [1].

Encapsulation efficiency (EE) in liposomal therapeutic formulations is considered a critical quality attribute since the treatment’s efficiency is usually strongly related to the amount of encapsulated drug. Furthermore, the presence of free drug may result in an undesired biodistribution, thereby causing toxic effects [7–9]. The determination of the encapsulated drug concentration can be accomplished by indirect or direct approaches. In the indirect method, the encapsulated drug is calculated by the subtraction of the free drug from the total drug concentration, whilst in the direct method, liposome isolation is necessary to directly determine the encapsulated drug concentration [7, 8]. In both strategies, the determination of EE requires the separation of the free drug (non-encapsulated) from the drug contained in the liposomes (encapsulated). Several procedures have been previously described for this purpose based on their size differences, such as dialysis membranes, ultracentrifugation, ultrafiltration, solid-phase extraction (SPE) and size exclusion chromatography (SEC) [8–11]. On the other hand, a methodology that effectively disrupts the liposomes and solubilises the released drug is necessary to determine the encapsulated drug concentration. The addition of alcohols, such as isopropanol (IPA), ethanol or methanol, to the liposome suspension has been previously described for this purpose [12]. However, some alcohols have been reported to cause protein precipitation, making them not suitable for peptide-based drugs [13]. Liquid-liquid extraction procedures based on the Bligh and Dyer or Folch classical methods have also been described for the extraction of lipids from animals and plant tissues [14, 15], biological fluids [16] or liposomes [17]. However, only a limited number of these procedures were applied for the extraction of proteins or peptides loaded in liposomes [18]. This methodology allows liposome lysis and subsequent well solubilisation of liposome components: lipids in the organic phase and peptide in the aqueous phase [15]. After free antigen separation from liposomes, a quantitative analysis technique is required to determine both free and total (or encapsulated) concentrations for assessing EE (%). The most widely employed separation technique is reversed-phase high-performance liquid chromatography (RP-HPLC), followed by capillary electrophoresis (CE) and field-flow fractionation (FFF). Depending on the drug’s nature, other techniques such as spectrophotometry, fluorescence spectroscopy and ^1^H and 2D nuclear magnetic resonance (NMR) have also been applied [9, 10, 12].

PS-Liposomes encapsulating autoantigenic peptides with different physicochemical properties were used in this study to evaluate several methods for EE determination (Table 1). When encapsulated within PS-Liposomes, the four tested antigenic peptides may promote immune tolerance against different AIDs: type 1 diabetes (T1D), celiac disease (CD), myasthenia gravis (MG) and multiple sclerosis (MS). Firstly, different protocols for liposome lysis and subsequent total or encapsulated peptide quantification were studied. On the other hand, different centrifugal filters (Amicon and Microcon) and the effect of filter passivation with bovine serum albumin (BSA) or polyethylene glycol (PEG) were evaluated for free peptide separation from PS-Liposomes. Moreover, an ultracentrifugation approach was also tested. The best conditions for indirect EE determination of each antigenic peptide were selected. Finally, the direct approach for EE determination was also evaluated by using insulin-loaded liposomes as model formulation. In this case, Vivaspin and Centrisart filters were used to improve isolation and recovery of PS-Liposomes, and the results were compared with the ones obtained by the indirect approach. Identification and quantification of autoantigenic peptides was performed by reversed-phase high-performance liquid chromatography with ultraviolet detection (HPLC-UV).

**Table 1:** Main physicochemical properties of the four antigenic peptides studied in this work

Properties	33-mer	Insulin	AChR	mMOG
Molecular weight (g·mol^−1^)	3913.49	5795.69	2182.44	2581.00
pI	3.1	5.3	4.4	10.7
Net charge (pH 7)	−2.0	−2.1	−1.0	3.1
Average hydrophilicity	−0.4	−0.5	−0.1	−0.1
Main attribute	Acidic	Neutral	Acidic	Basic
Hydrophobic residues (%)	60.6	33.3	40.0	38.1
Acidic residues (%)	6.1	7.8	15.0	4.8
Basic residues (%)	0	7.8	5.0	23.8
Neutral residues (%)	33.3	51.0	40.0	33.3

## Experimental section

### Materials and solutions

Acetonitrile (ACN, HPLC grade), chloroform (CHCl_3_), hydrochloric acid (HCl), trifluoracetic acid (TFA) and acetic acid were sourced from Sigma-Aldrich (Darmstadt, Germany). Methanol (MeOH, LC/MS grade) and isopropanol (IPA, LC/MS grade) were both acquired from Honeywell (Seelze, Germany). Dulbecco’s phosphate-buffered saline (DPBS) with Ca^+^/Mg^+^ was purchased from Cytiva (Logan, UT). Triton X-100 was purchased from Fisher Scientific (Waltham, MA, USA). Antigenic peptides (33-mer peptide from alfa-gliadin, insulin, murine myelin oligodendrocyte glycoprotein (mMOG) and acetylcholine receptor (AChR)) were provided by the Peptide Synthesis Facility of the Department of Medicine and Life Sciences of the Pompeu Fabra University (Barcelona, Spain), with a purity exceeding 90%. All autoantigenic peptide standard stock solutions were prepared from the lyophilised peptides at a concentration of 1000 mg·L^−1^. Both 33-mer and mMOG peptides were dissolved in water, AChR in DPBS and insulin in water with 2% of acetic acid. Calibration standards were prepared daily by diluting with each solvent the stock solution to the calibration range (10–250 mg·L^−1^).

### Liposome formulation manufacturing

PS-Liposome formulations were manufactured by a solvent-injection method in a cleanroom facility at Ahead Therapeutics (Arboç, Catalonia, Spain). Liposomes were composed of phosphatidylcholine (PC, Lipoid), phosphatidylserine (PS, Lipoid) and cholesterol (CHOL, Merck). Lyophilised lipids were dissolved in ether at 55 °C, and the lipid solution was rapidly injected into the antigenic peptide solution (dissolved in DPBS). Finally, PS-Liposomes were extruded using a Lipex® Extruder (Evonik) and dialysed for 24 h, resulting in a milky homogeneous suspension.

### Total peptide determination

A method that enables complete liposome lysis and extraction of the encapsulated peptide is required to determine either encapsulated or total (free plus encapsulated) drug concentration. Only two methods allowed a successful PS-Liposome disruption (transparent solution). The first method consists of a liquid-liquid extraction procedure previously described [19], based on the classical Bligh and Dyer lipid extraction method. Briefly, 250 µL of MeOH and 125 µL of CHCl_3_ were added to 100 µL of liposome sample. After stirring, 250 µL of 0.1 M HCl and 125 µL of CHCl_3_ were added, and the solution was stirred again. The use of DPBS instead of 0.1 M HCl was also studied for AChR peptide. Centrifugation at 100 × g for 5 min at room temperature was performed to allow a better phase separation. Finally, 100 µL of the upper MeOH-water phase, which contains the extracted autoantigenic peptide, was collected. On the other hand, the second method involves a direct liposome lysis by adding 50% of isopropanol to the PS-Liposomes suspension. The solution was then centrifuged at 14,000 × g for 10 min at room temperature and the supernatant, which contains the extracted peptide, was collected. Spiked placebo PS-Liposomes with each peptide at a concentration of 200 mg·L^−1^ were used to evaluate recoveries. Both methods were tested with the four antigenic peptides, and their quantification was performed afterwards by HPLC-UV.

### Free peptide and liposome separation

The separation of the unencapsulated peptide (free) from liposomes is required to determine both free and encapsulated peptide concentrations. For free peptide determination, the use of two different centrifugal filters (Amicon Ultra (100 kDa) and Microcon YM30 (30 kDa)) and an ultracentrifugation approach were evaluated. In the case of both Amicon Ultra and Microcon filters, 200 μL of peptide stock solutions at 200 mg·L^−1^, or placebo PS-Liposomes spiked with each peptide stock solution at a final concentration of 200 mg·L^−1^, was loaded to the filter and centrifuged at 14,000 × g for 10 min at room temperature. The filtration residue was then rinsed twice by adding 100 µL of water or DPBS and centrifuging under the same conditions. The filtrates were pooled together, and sufficient water was added to reach the final volume of 400 µL. For Amicon Ultra devices, filter passivation was also tested by using bovine serum albumin (BSA) and polyethylene glycol (PEG) as described elsewhere [20]. Briefly, 500 μL of a 1% m/v BSA or 5% v/v PEG solution was added to the filter, capped and left for 24 h at room temperature. Afterwards, the passivation solution was removed, and the filter was thoroughly rinsed with tap water. A volume of 500 μL of distilled water was then added to the filter and centrifuged at 14,000 × g for 30 min. Residual water was removed by inverting the filter in a vial and spinning at 900 × g for 2 min. For the ultracentrifugation approach, 200 µL of placebo PS-Liposomes spiked with each peptide stock solution at a final concentration of 200 mg·L^−1^ was centrifuged at 4 °C for 40 min at 14,000 × g, and supernatant was collected. The effect of water rinses was tested by adding 100 µL water or DPBS to 100 µL of the collected supernatant and centrifuging it for 20 additional minutes under the same conditions. The obtained supernatant was pooled with the first one and the process was repeated one more time. Finally, the collected filtrates from the centrifugal filters and supernatants from the ultracentrifugation method were analysed by HPLC-UV to determine free peptide concentration.

For encapsulated peptide determination, Amicon Ultra (100 kDa) and Microcon YM30 (30 kDa) centrifugal filters were also used. Additionally, Vivaspin 500 (100 and 300 kDa) and Centrisart I (300 kDa) were evaluated. In this case, PS-Liposomes with encapsulated peptide are necessary to test the different filters, and insulin-loaded PS-Liposomes were employed as a model formulation. For Amicon Ultra, Microcon and Vivaspin, 100 μL of insulin-loaded PS-Liposomes and 100 μL of DPBS were mixed, loaded to each filter and centrifuged at 14,000×g for 10 min at room temperature. The filtration residue was rinsed twice as previously described, and liposomes were recovered from the upper reservoir by upside-down centrifugation in a new vial (3 min at 900 × g). For Centrisart I devices, 500 μL of insulin-loaded PS-Liposomes and 500 μL of DPBS were mixed and loaded to the centrifuge tube (outer tube), after removing the floater (inner tube). After reinserting the floater, the centrifuge tube was allowed to stand for 5 min to ensure complete membrane wetting and centrifuged at 2500 × g for 30 min at room temperature, following manufacturer instructions. Then, the floater was rapidly removed, and the concentrated liposomes were obtained in the centrifuge tube. Liposome lysis with 50% isopropanol was then applied to each obtained tube before HPLC-UV analysis.

On the other hand, size exclusion chromatography (SEC) was used to study mMOG peptide interaction with PS-Liposomes. For this purpose, Micro Bio-Spin P-6 column from Bio-Rad with a 6 kDa size exclusion was used. A volume of 50 µL of mMOG stock solution at 500 mg·L^−1^ was loaded in one spin column and centrifuged at 1000 × g for 4 min. The obtained filtrate was analysed by HPLC-UV. The same procedure was applied to 50 µL of placebo PS-Liposomes spiked with mMOG solution at a final concentration of 500 mg·L^−1^. In this case, liposome lysis with 50% isopropanol was applied to the filtrate before HPLC-UV analysis.

### Encapsulation efficiency (EE(%)) determination

Encapsulation efficiency can be assessed using either an indirect or direct approach. The indirect method involves quantifying the free drug concentration, after separating it from the liposomes, and the total drug concentration. Total drug concentration (free plus encapsulated drug) can be quantified after liposome disruption and extraction of the encapsulated drug. The indirect EE is calculated as: EE(%) = (Total drug concentration − Free drug concentration)/Total drug concentration. On the other hand, the direct strategy involves separating the liposomes from the free drug and allowing the disruption of the isolated liposomes to solely determine the encapsulated drug concentration. The direct EE is calculated as: EE(%) = Encapsulated drug concentration/Total drug concentration.

### Peptide quantification by HPLC-UV

Separation and quantification of antigenic peptides was performed by high-performance liquid chromatography with ultraviolet-visible detection (HPLC-UV) on an UHPLC 1290 Infinity system (Agilent Technologies, Waldbronn, Germany). Chromatographic separation was carried out using a Zorbax 300SB-C3 column (3.5 µm particle diameter, 300 Å pore diameter, 150 × 4.6 mm total length (LT) × internal diameter (ID), Agilent Technologies). A gradient elution method was employed with water (solvent A) and ACN (solvent B) both containing 0.1% v/v TFA, at a flow rate of 0.5 mL·min^−1^. The optimised gradient of solvent B was as follows: from 5 to 100% (v/v) (0–10 min, linear), 100% (v/v) (10–15 min), from 100 to 5% (v/v) (15–18 min, linear) and 5% (v/v) (18–25 min). The column’s temperature was maintained at room temperature throughout the analysis. A 5 µL injection volume was used, and the autoantigen peptides were detected based on their UV absorbance at a wavelength of 280 nm. The RP-HPLC-UV method was validated for each antigen peptide in terms of linearity, sensitivity, accuracy, precision and specificity. Method linearity was assessed using peptide standard solutions (in water for 33-mer and mMOG, in DPBS for AChR and in water with 2% of acetic acid for insulin). Method sensitivity was evaluated by determining the limits of detection (LOD) and quantification (LOQ) by visual inspection using serial dilutions of the standard peptide solutions. Intra-day and inter-day accuracy and precision were evaluated as recoveries and relative standard deviation (%RSD), respectively, analysing peptide solutions at three concentration levels (20, 80 and 150 mg·L^−1^) in triplicate on the same day (*n* = 3) and on three different days (*n* = 3). Specificity was assessed by applying free and total antigen determination protocols to placebo (empty) PS-Liposomes to evaluate potential matrix interferences. Furthermore, carryover was evaluated using water blank injections.

### Peptide identification by HPLC-MS

The protocol for antigenic peptide identification by liquid chromatography coupled to mass spectrometry (HPLC-MS) is provided in the Supplementary Material.

### Zeta potential determination by dynamic light scattering (DLS)

The protocol for zeta potential measurements by dynamic light scattering (DLS) is provided in the Supplementary Material.

## Results and discussion

### Total peptide determination. Liposome lysis

Four antigenic peptides (33-mer, insulin, mMOG and AChR) with different physicochemical properties (Table 1) were selected for this study. Peptide quantification was carried out by reversed-phase high-performance liquid chromatography with ultraviolet detection (RP-HPLC-UV), and the method was validated for each peptide in terms of linearity, sensitivity, intra-day and inter-day accuracy and precision, and specificity. Excellent linearity was obtained for all peptides (*R*^2^ > 0.999), with linearity ranges between 1 and 250 mg·L^−1^, except for mMOG (3.0–250 mg·L^−1^), and a working range of 10–250 mg·L^−1^. Method sensitivity showed LOD and LOQ values of 0.5 and 1 mg·L^−1^, respectively, for all peptides, except for mMOG (2.5 and 3.0 mg·L^−1^). Intra- and inter-day accuracy and precision, evaluated at 20, 80 and 150 mg·L^−1^, yielded recoveries between 98 and 102% and %RSD values below 3% in all cases (Table S-1). The method enables effective separation and accurate quantification of the antigenic peptides in the presence of lipids and degradation products, allowing precise EE determination. These results, together with the presence of UV-absorbing chromophores in the studied peptides, further demonstrate the suitability of the HPLC-UV technique for determining EE in liposomal formulations. Moreover, the technique represents a cost-effective and reproducible alternative to more complex techniques, such as LC-MS, for routine quality control, batch release and stability studies. On the other hand, although no additional peaks were observed by HPLC-UV at the corresponding retention times of the selected antigen peptides (3.8 min for mMOG, 4.3 min for 33-mer, 4.1 min for insulin and 6.7 min for AChR) (Figure S-1), peptide identity was further confirmed by high-performance liquid chromatography coupled to mass spectrometry (HPLC-MS), as described in the Supplementary data (Figures S-2, S-3, S-4, S-5).

**Fig. 1:**
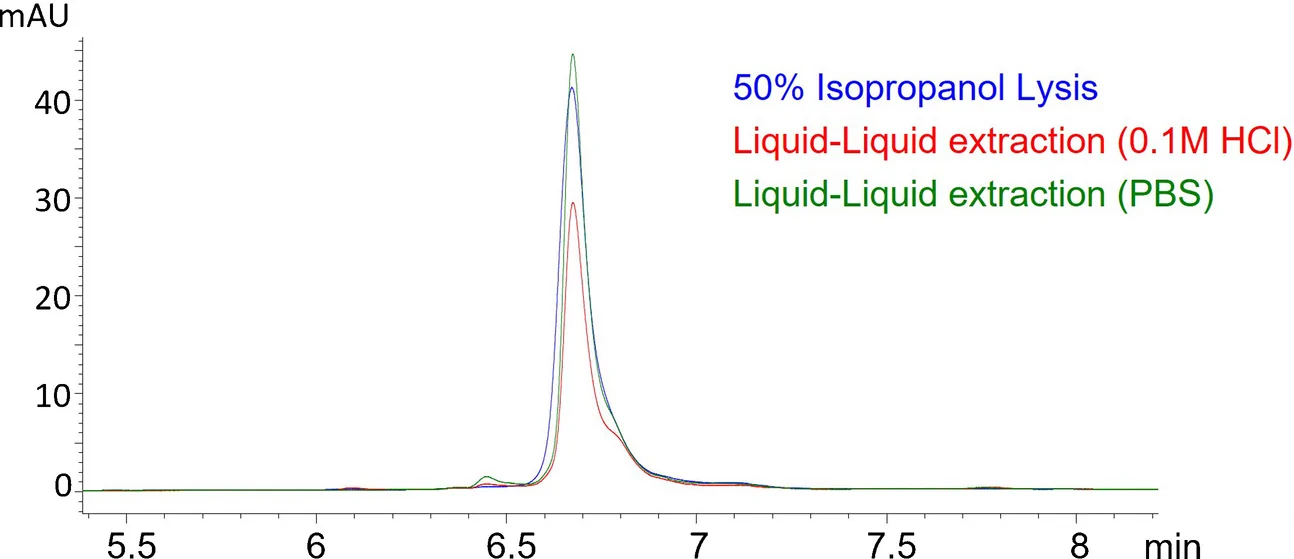
HPLC-UV chromatograms obtained for placebo PS-Liposomes spiked with AChR (200 mg·L^−1^) after applying the different protocols for liposome lysis and total peptide determination

Methodologies allowing complete liposome lysis were evaluated for total antigenic peptide determination in PS-Liposomes. In this regard, different proportions of organic solvents such as methanol, isopropanol or acetonitrile [12], as well as the addition of detergent Triton X-100 were tested, following previously described methods for liposome lysis [21, 22]. Moreover, a liquid-liquid extraction procedure based on the Bligh-Dyer classical method was also applied [18]. However, methanol and acetonitrile do not allow complete PS-Liposome lysis, although high amounts were added, resulting in a turbid solution. Similarly, it was not possible to achieve PS-Liposome lysis by the addition of different percentages of Triton X-100. However, both the addition of 50% of isopropanol and the liquid-liquid extraction procedure allowed successful PS-Liposome lysis, resulting in a completely transparent solution. Therefore, these two methods were applied to spiked placebo PS-Liposomes with the different antigenic peptides at 200 mg·L^−1^. As shown in Table S-2, the recoveries obtained for both methods were similar in all cases. Best results were achieved by using the liquid-liquid extraction for 33-mer and mMOG, whilst 50% of isopropanol was optimal for insulin. In the case of AChR, recoveries below 90% were obtained when using the previously described methods, prompting modifications to the liquid-liquid extraction procedure. Due to the better solubility of this peptide in DPBS buffer, the addition of 0.1 M HCl was replaced in the protocol for DPBS (Sect. "Total peptide determination"). Applying this modification, complete liposome lysis is maintained and a 98% of peptide recovery was achieved. By way of comparison, Fig. 1 shows the chromatograms obtained for AChR when applying the different protocols for total peptide determination. Finally, HPLC-UV method specificity was evaluated by applying both liquid-liquid extraction and 50% of isopropanol lysis protocols to placebo (empty) PS-Liposomes, revealing no lipid-derived interferences. Additionally, carryover was negligible, as confirmed with water blank injections (Figure S-6). Matrix interferences and fortified specificity were assessed by applying the selected total antigen determination protocol to placebo PS-Liposomes spiked at three quality control levels (20, 80 and 150 mg·L^−1^), yielding recoveries above 99% in all cases (Table S-3).

### Separation and determination of free peptide. Indirect method

**Fig. 2:**
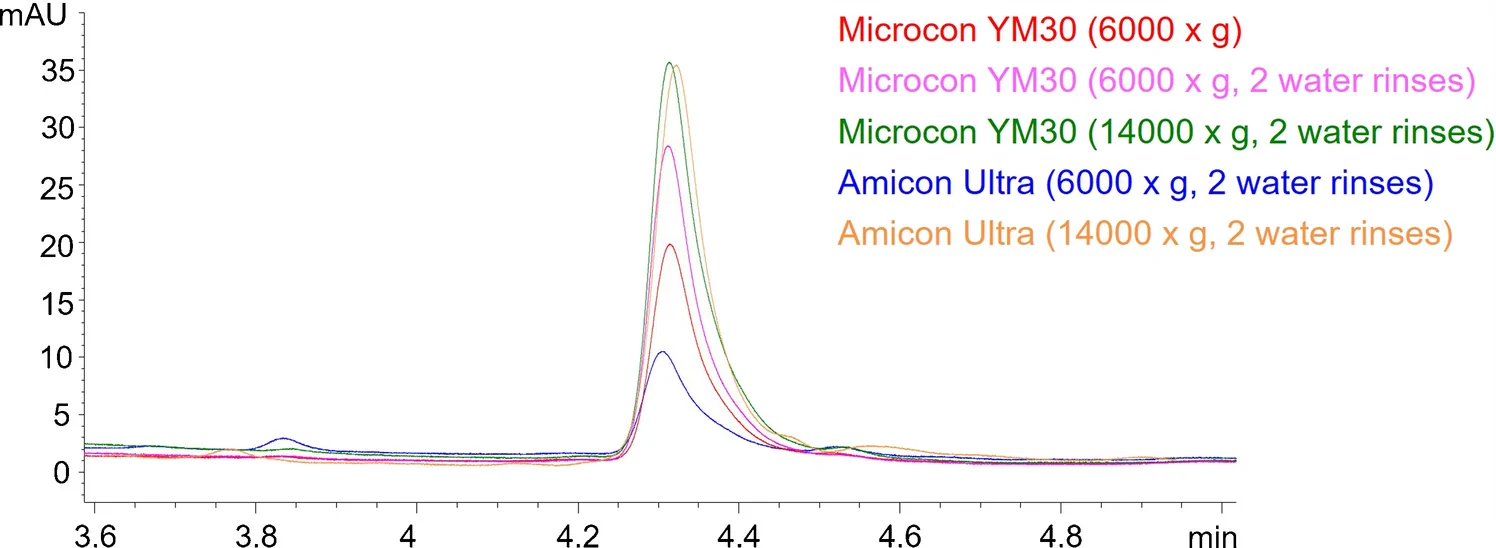
HPLC-UV chromatograms obtained for the analysis of the filtrates obtained by using Microcon YM30 and Amicon Ultra centrifugal filters under different conditions for placebo PS-Liposomes spiked with 33-mer peptide (200 mg·L^−1^)

The encapsulation efficiency (EE (%)) can be determined following either indirect or direct approaches. In this study, the indirect strategy was firstly applied, as it is the most widely used methodology. This preference is due to the challenges associated with achieving good liposome recovery after free drug removal when using the direct approach. With this aim, three different methods for the separation and determination of free antigenic peptides from PS-Liposomes were evaluated: a horizontal centrifugal filter (Microcon YM30, 30 kDa), an upright centrifugal filter (Amicon Ultra, 100 kDa) and an ultracentrifugation approach. Spiked placebo PS-Liposomes with each antigenic peptide (33-mer, insulin, mMOG and AChR) at a final concentration of 200 mg·L^−1^ were used to evaluate the recoveries. For both centrifugal filter evaluations, two different centrifugal speeds (6000 × g and 14,000 × g) and the addition of filter rinses with water were tested. Figure 2 shows, by way of example, the chromatograms obtained by HPLC-UV for the analysis of the filtrates under each condition using placebo PS-Liposomes spiked with 33-mer peptide. As can be observed, free antigen recoveries increased with the centrifugation speed and the addition of water rinses. Similar results were obtained for both centrifugal filters using the best conditions (14,000 × g and 2 water rinses), despite the differences in the cut-off sizes of both filters. Analogous results were also obtained for the other peptides. However, the Microcon YM30 filters offered low reproducibility as they were easily clogged by liposomes, despite attempts to enhance filtration through larger sample dilutions. Therefore, the use of Amicon filters at 14,000 × g for 10 min with two water rinses was considered more suitable. Using these conditions, recoveries greater than 75% were obtained for 33-mer and insulin peptides, whilst AChR and mMOG antigen recoveries were below 5% (Table 2). To enhance recoveries and prevent partial peptide adsorption on filters, the effect of filter passivation was studied using 1% BSA and 5% PEG. Spiked placebo PS-Liposomes with each peptide at 200 mg·L^−1^ were loaded on BSA passivated filters, and higher recoveries were yielded for 33-mer and insulin (Table 2). However, AChR and mMOG peptide recoveries remained very low. In the case of AChR, recoveries close to 0% were also obtained when only peptide solutions were filtered, indicating strong adsorption of AChR onto the filter. Therefore, determination of free peptide by centrifugal filtration was not considered suitable in this case. In contrast, for mMOG, recoveries close to 100% were obtained when peptide solutions were filtered whilst peptide recovery dropped to < 5% when using spiked placebo PS-Liposomes. This fact pointed out the possibility of mMOG peptide adsorption on PS-Liposomes external membrane.

**Table 2:** Recoveries of the free peptide determination when using Amicon’s centrifugal filters and the ultracentrifugation approach with a spiked placebo PS-Liposomes with each peptide at 200 mg·L^−1^

Recoveries (%)		Spiked placebo PS-Liposomes with peptide (200 mg·L^−1^)			
		**33-mer (CD)**	**Insulin (T1D)**	**AChR (MG)**	**mMOG (MS)**
Amicon Ultra (100KDa)	**Non-passivated**	76%	79%	0%	< 5%
	**BSA passivated**	88%	80%	0%	< 5%
Ultracentrifugation(14,000 × g)	**4°C, 40 min**	100%	95%	109%	47%
	4°C, 20 min, and H_2_O/DPBS rinses (×2)	82%	81%	78%	31%

**Fig. 3:**
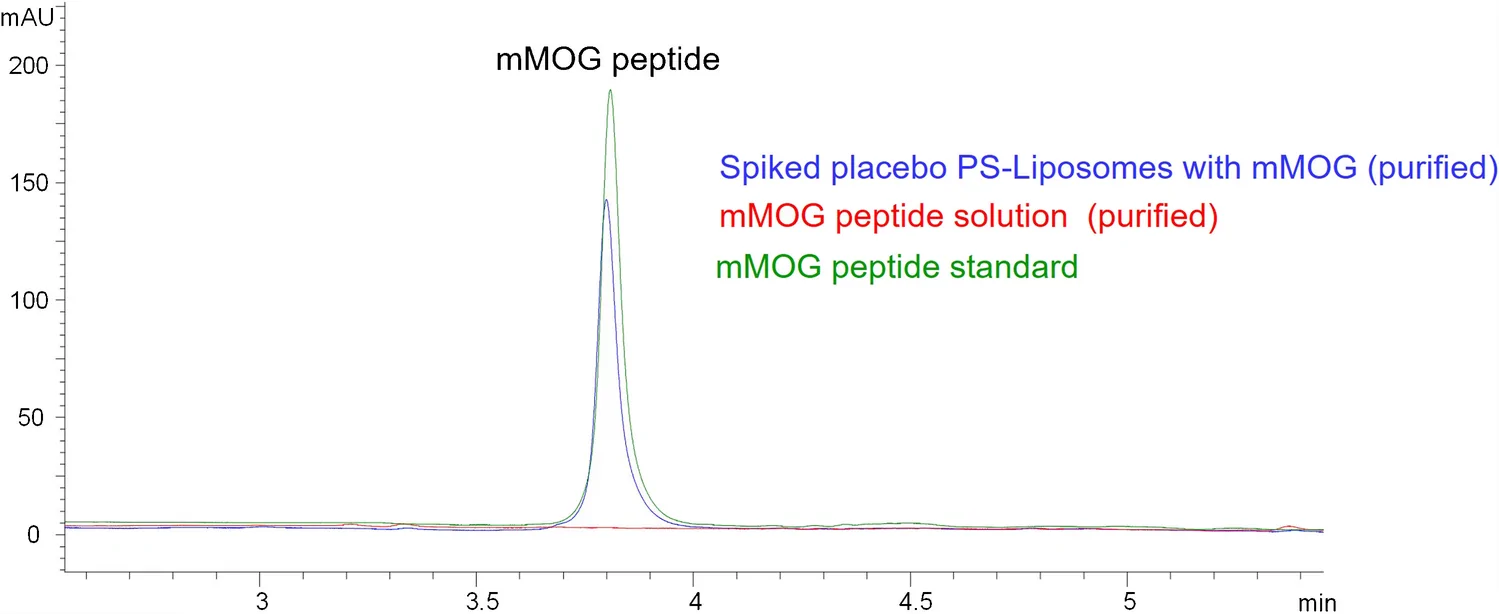
Chromatograms obtained for the analysis by HPLC-UV of the filtrates obtained by using a 6 kDa size exclusion column for mMOG peptide solution and spiked placebo PS-Liposomes with mMOG (500 mg·L^−1^), with respect to mMOG peptide standard

In order to study the possible mMOG peptide adsorption on PS-Liposomes, a 6 kDa size exclusion column (Micro Bio-Spin P-6, Bio-Rad) was used. To this end, a mMOG peptide solution and a spiked placebo PS-Liposome with mMOG (500 mg·L^−1^) were loaded in two different columns, and both filtrates were analysed by HPLC-UV. In the case of spiked placebo PS-Liposomes with mMOG, liposome lysis was applied before analysis. As shown in Fig. 3, when only the mMOG peptide solution was loaded to the column, the peptide is retained in the gel and the mMOG peak was not detected by HPLC-UV. In contrast, when spiked placebo PS-Liposomes were loaded, mMOG was eluted with liposomes (> 6 kDa exclusion limit) and the mMOG peak was detected by HPLC-UV. These results could explain the poor free mMOG recoveries (Table 2) due to electrostatic interactions of the peptide with placebo PS-Liposomes. PS-Liposomes exhibit zeta potential values, determined by dynamic light scattering (DLS) as detailed in the Supplementary Material, ranging from −20 to −25 mV, indicating a negatively charged external membrane. Unlike the other studied peptides, mMOG presents a positive charge (mMOG’s pI of 10.7 is higher than the medium pH), which may cause its electrostatic adsorption onto the negatively charged liposomal surface. Importantly, encapsulation in liposomes is not restricted to entrapment within the aqueous core (hydrophilic drugs); depending on the physicochemical properties of the peptide, it may also localise within or be strongly associated with the phospholipid bilayer (lipophilic or amphiphilic drugs). This is particularly relevant for PS-Liposomes, whose mechanism of action relies on their rapid recognition and uptake by macrophages and dendritic cells after entering the bloodstream. In this context, an API associated with the external membrane is not functionally “free”; rather, it is co-delivered with the liposome to target immune cells. Therefore, as long as the membrane-associated API remains stably bound and co-migrates with the vesicles during separation procedures, it behaves biologically as an encapsulated fraction, contributing directly to the intended immunological effect. Therefore, for further studies, the fraction of mMOG electrostatically adsorbed onto the PS-Liposomes external membrane will be also considered as encapsulated antigen.

On the other hand, the ultracentrifugation approach was also assessed using spiked placebo PS-Liposomes with each peptide (200 mg·L^−1^). Different centrifugation times and rinsing steps were tested. Centrifugation speed was maintained at 14,000 × g based on the previous results with centrifugal filters, and temperature was set up at 4 °C to avoid peptide degradation. In the case of AChR, rinses were conducted with DPBS instead of water due to its better solubility in that medium. The ultracentrifugation without rinses for a longer time (4°C for 40 min at 14,000 × g) provided recoveries above 95% for all autoantigen peptides, except for the mMOG antigen (Table 2). The introduction of two rinses, by adding water or DPBS and centrifuging for 20 min at 14,000 × g at 4 °C each time (40 min in total), did not improve free peptide recoveries in any case (Table 2). In the case of mMOG, rinses with both water (31%) and DPBS (33%), as well as centrifugation at room temperature (29%), were also tested to study possible differences in mMOG peptide adsorption on PS-Liposomes external membrane. However, the highest free peptide recovery was also obtained by centrifuging at 4 °C for 40 min (47%). Taking into account all the obtained results, the best free antigen recoveries were provided with the ultracentrifugation method for 40 min, without rinses, by ensuring the complete precipitation of liposomes and allowing all free antigen isolation (Table 2). In addition, to evaluate matrix interferences in free antigen determination, ultracentrifugation was applied to placebo (empty) PS-Liposomes, revealing no lipid-derived interferences (Figure S-6).

To further confirm the results obtained with spiked PS-Liposomes, the most promising methodologies for free peptide determination (BSA passivated Amicon filters and ultracentrifugation) were applied to PS-Liposome formulations with encapsulated peptides. The encapsulation efficiency (EE (%)) of each batch was calculated relative to the total antigen concentration determined for each peptide by the previously selected method. The obtained results are shown in Table 3. When comparing the EE (%) obtained within the same batch using the different methodologies, it can be observed that the lowest values were obtained using the ultracentrifugation method for 40 min in all cases. These results are in accordance with the previously obtained recoveries in spiked placebo samples. Higher recoveries in free peptide determination for spiked placebo samples are associated with lower EE (%) in the analysed PS-Liposomes formulations. Therefore, the most reliable method for free peptides determination was considered the ultracentrifugation at 14,000 × g for 40 min at 4 °C in all cases. In the case of mMOG, the highest EE (%) was obtained, probably due to its tendency to adsorb on the PS-Liposomes external membrane. Ultracentrifugation, relying on density-based sedimentation rather than molecular weight cut-offs or polymeric membranes, avoids peptide-membrane adsorption and enables reliable separation of free peptide from liposome-associated peptide. In contrast, centrifugal filters often exhibit adsorption effects, especially with hydrophobic or amphiphilic peptides, leading to low recovery, poor mass balance and limited reproducibility. Although ultracentrifugation may require longer run times, it provides a more robust and reproducible approach for routine EE (%) determination in peptide-loaded liposomal formulations. Finally, the optimal conditions for EE (%) determination by the indirect approach were applied to other batches of these PS-Liposome formulations. Table S-4 shows the EE (%) results obtained for each batch applying the ultracentrifugation method for free peptide determination and the selected method for each peptide for total peptide determination. As can be observed, different EE (%) values were obtained for each encapsulated peptide on PS-Liposomes. This fact could be attributed to the different physicochemical properties that each peptide presents. In hydrophobic peptides containing large percentages of hydrophobic residues and less than 25% of the charged ones (i.e. the sum of acidic and basic residues), such as 33-mer, its encapsulation is the lowest. Whilst in hydrophilic peptides, such as mMOG, containing more than 25% of charged residues, the EE (%) is much higher. It could be related to the tendency of hydrophobic drugs to be encapsulated within the membrane’s lipid bilayer, whereas the hydrophilic drugs are encapsulated in the aqueous core [23]. In addition, the positive charge of mMOG peptide in the medium pH (pI > pH) can promote electrostatic interactions with negatively charged PS-Liposomes, as discussed above, increasing even more its encapsulation. Differences in the EE (%) between batches of PS-Liposome formulations encapsulating the same peptide (Table 3 vs. Table S-4) are attributed to minor modifications applied during the liposomes manufacturing of each batch.

**Table 3:** Encapsulation efficiency (EE (%)) results obtained when different free peptide determination methodologies were applied to PS-Liposome formulations encapsulating different antigens. EE (%) of each batch is calculated with respect to the determined total antigen concentration

EE (%)		Batch 1 33-mer (CD)	Batch 2 Insulin (T1D)	Batch 3 AChR (MG)	Batch 4 mMOG (MS)
Amicon Ultra (100 kDa)	**BSA passivated**	41%	52%	-	90%
Ultracentrifugation (14,000 × g)	**4**°**C, 40 min**	**31%**	**48%**	**62%**	**69%**
	**4**°**C, 20 min and H**_**2**_**O rinses (× 2)**	43%	62%	71%	70%

### Isolation of encapsulated liposomes. Direct method

After establishing the optimal methods for determining both free and total peptide concentrations in PS-Liposomes, various methodologies for the direct encapsulation efficiency (EE) (%) determination were evaluated. Insulin-loaded PS-Liposomes (batch 5) were used as a model formulation, as liposomes with encapsulated peptide were necessary to assess the direct approach.

Firstly, the most promising methods for free peptide determination were applied (i.e. ultracentrifugation and Amicon Ultra filters) for both approaches. The obtained results are shown in Table 4. Taking into account the previous recoveries for free peptide determinations, the EE (%) of 59% obtained by ultracentrifugation can be considered the most reliable value for this insulin-loaded formulation (batch 5). However, very low EE (%) was obtained by using this methodology in the direct approach (Table 4), pointing out that the complete recovery of sedimented liposomes was not possible. In addition, Amicon Ultra filters also provided lower EE (%), demonstrating poor recovery of liposomes, probably due to adsorption on the filter. Thus, other upright centrifugal filters (Vivaspin), with different cut-offs, and Centrisart I devices were also tested to improve the recoveries of encapsulated liposomes in the direct method. With Vivaspin filters, liposomes get also trapped on the filter, leading to their incomplete recovery and resulting in inaccurately low EE (%), as can be observed in Table 4. Only with Centrisart I devices, which work in the opposite direction to centrifugal force preventing premature filter blockage, an encapsulation efficiency of 58% was obtained, very similar to the reference value obtained by the indirect ultracentrifugation approach (59%). On the other hand, the indirect method using all the filter devices was also applied to batch 5 to compare the different indirect EE (%) values obtained. In this regard, we can conclude that the Amicon Ultra 100 kDa and Vivaspin 300 kDa provided slightly lower EE (%), which could indicate certain peptide leakage due to strong conditions and water rinses. In contrast, very high EE (%) values were obtained by using Vivaspin 100 kDa and Centrisart I filters demonstrating incomplete free insulin recoveries.

**Table 4:** Indirect and direct EE (%) method comparison using insulin-loaded PS-Liposomes as model formulation

EE (%)	Batch 5 (insulin PS-Liposomes)	
	**Direct approach**	**Indirect approach**
Ultracentrifugation	11%	**59%**
Amicon Ultra (100 kDa)	28%	50%
Vivaspin 500 (100 kDa)	25%	79%
Vivaspin 500 (300 kDa)	24%	47%
Centrisart I (300 kDa)	58%	96%

Considering all these results, it can be observed that similar EE (%) results were obtained by both direct and indirect approaches using the appropriate method (Centrisart and Ultracentrifugation, respectively), demonstrating the results’ reliability. Nevertheless, the indirect approach using ultracentrifugation may be regarded as the most appropriate method for EE (%) determination in quality control of PS-Liposome formulations, as it can be considered more cost-effective and less time-consuming.

## Conclusions

In this study, several analytical approaches were compared to accurately determine the encapsulation efficiency (EE (%)) of different peptides in PS-liposome formulations, a critical quality attribute directly related to the treatment’s effectiveness. Liposome disruption methods showed that efficient liposome disruption and encapsulated drug extraction are highly dependent on both liposome composition and peptide physicochemical properties. Among the tested methods, the addition of 50% isopropanol and a Bligh and Dyer-based liquid-liquid extraction were the most effective.

For quantification of free peptides, several centrifugation filters and conditions were evaluated, including filter type, centrifugation parameters, rinsing steps, and membrane passivation. However, the ultracentrifugation approach consistently provided the highest and most reliable peptide recoveries, highlighting its robustness for separating free and encapsulated fractions. In the direct EE% determination approach, liposome isolation efficiency was strongly device-dependent, and most tested filters produced inconsistent results. Only Centrisart I devices lead to reliable results. Finally, it was proved that similar results can be obtained by both indirect and direct strategies after appropriate method selection. Overall, these findings offer practical guidance for selecting and optimising methodologies according to formulation characteristics, supporting the development of robust and reliable analytical methods for EE (%) determination.

## Supplementary Information

Below is the link to the electronic supplementary material.

Supplementary file1 (PDF 465 KB)

## Data Availability

The authors confirm that the data supporting the findings of this study are available within the article and its Supplementary material. Raw data that support the findings of this study are available from the corresponding author, upon reasonable request.
