# Supplementary material for: Encapsulation efficiency determination methods for the analysis of different antigenic peptides within phosphatidylserine-liposomes
Source: Anal Bioanal Chem. 2026 Mar 25;418(18):5993–6002. doi: 10.1007/s00216-026-06453-x (PMC13541897; doi:10.1007/s00216-026-06453-x)
Supplement: Supplementary file 1 [file 216_2026_6453_MOESM1_ESM.pdf]

SUPPLEMENTARY MATERIAL

ENCAPSULATION EFFICIENCY DETERMINATION METHODS FOR THE ANALYSIS OF DIFFERENT ANTIGENIC PEPTIDES WITHIN PHOSPHATIDYLSERINE-LIPOSOMES

Irene Latorre[1,2], Montserrat Mancera-Arteu[1*], Oumaima El Ouahabi[1,2], Míriam Salvadó[2], Sílvia Rodríguez-Vidal[2], Victoria Sanz-Nebot[1]

[1]Department of Chemical Engineering and Analytical Chemistry, Institute for Research on Nutrition and Food Safety (INSA·UB), University of Barcelona, Martí i Franquès 1-11, 08028 Barcelona, Spain

[2]Ahead Therapeutics SL, Barcelona, Spain

*Corresponding author: montsemancera@ub.edu (M. Mancera-Arteu, PhD)

Supplementary Experimental

Peptide identification by HPLC-MS

The four antigenic peptides were identified using a 1260 Infinity liquid chromatograph (Agilent Technologies, Waldbronn, Germany) coupled to a 6220 oa-TOF LC/MS mass spectrometer with an orthogonal G1385−44300 interface (Agilent Technologies). Chromatographic separation was performed on a Zorbax 300SB-C_3 column (3.5 µm particle diameter, 300 Å pore diameter, 150x4.6 mm total length (LT) × internal diameter (ID), Agilent Technologies), employing gradient elution at a flow rate of 0.35 mL/min. Mobile phases consisted of water (A) and acetonitrile (B), both containing 0.1% (v/v) of formic acid (FA). The gradient elution for solvent B was: 5% to 100% (v/v) (0-10 min), 100 % (v/v) (10–15 min), from 100 % to 5 % (v/v) (15–18 min) and 5 % (v/v) (18–25 min). The injection volume was 20 µL. MS analysis was performed in positive ion mode under the following conditions: capillary voltage 4000 V; drying gas (N_2) flow rate 8L·min^{-1} and temperature 350 ºC; nebulizer gas (N_2) pressure 30 psig; fragmentor voltage 325 V; skimmer voltage 80V; and OCT 1 RF Vpp voltage 250V. Data were acquired in profile mode at 1 spectrum·s^{-1} over an m/z of 100 -3200 using at the highest resolution mode (4GHz).

Zeta Potential determination by Dynamic light scattering (DLS)

The nanoparticle surface charge is expressed as zeta potential and determined by Dynamic light scattering (DLS). DLS measurements were performed using a Zetasizer Nano ZS instrument (Malvern Instruments, Malvern, UK), applying the diffusion barrier method. The capillary cell of a disposable Micro-Cuvette (DTS1070, Malvern Panalytical) was firstly filled with DPBS. Subsequently, 100 µL of the PS-Liposome sample was injected directly into the bottom of the cell, ensuring that the sample is

properly settled and centered at the bottom of the capillary cell. Air bubbles should be avoided for proper measurement. Each sample was analysed in triplicate in automatic run mode.

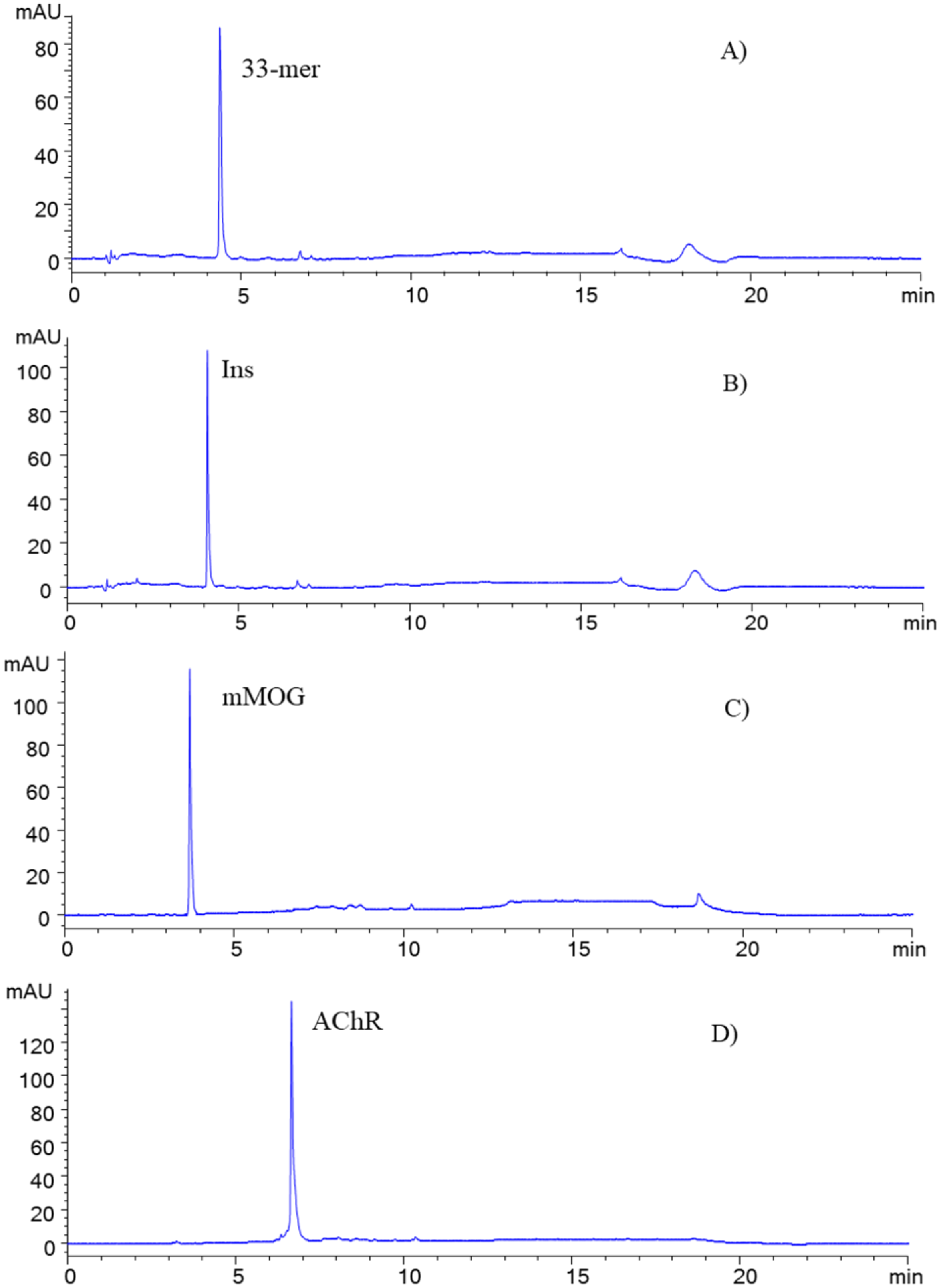

Figure S-1.- HPLC-UV chromatograms obtained for each peptide at a concentration of 100 mg·L^{-1}: A) 33-mer (4.3 min), B) Insulin (4.1 min), C) mMOG (3.8 min), D) AChR (6.7 min).

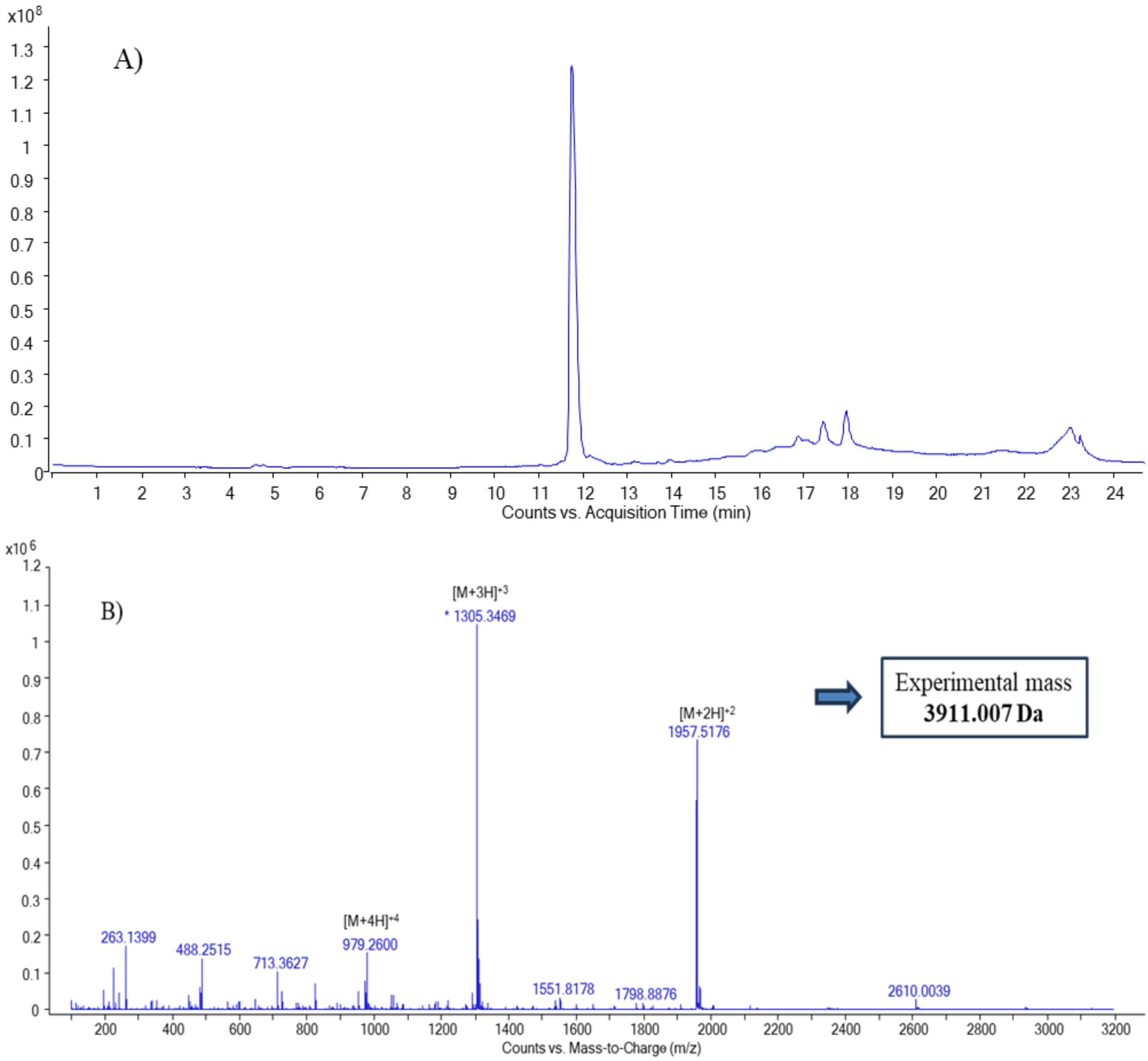

Figure S-2.- A) Extracted ion chromatogram of 33-mer peptide (50 mg·L^{-1}) obtained by HPLC-MS. B) Mass spectrum of the peak eluting at 11.8 min.

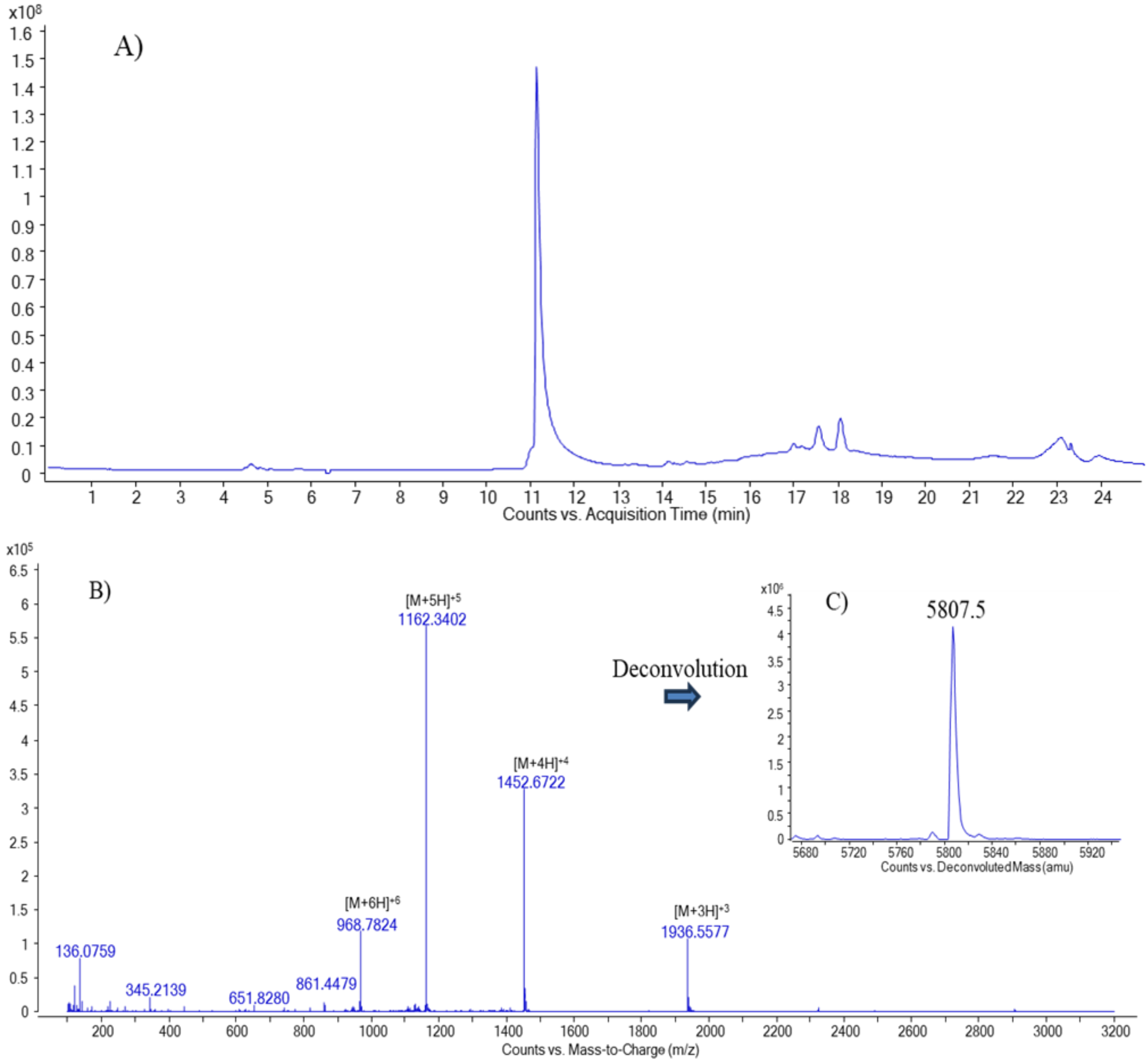

Figure S-3.- A) Extracted ion chromatogram of Insulin peptide (50 mg·L^{-1}) obtained by HPLC-MS. B) Mass spectrum of the peak eluting at 11.1 min. C) Deconvoluted mass spectrum.

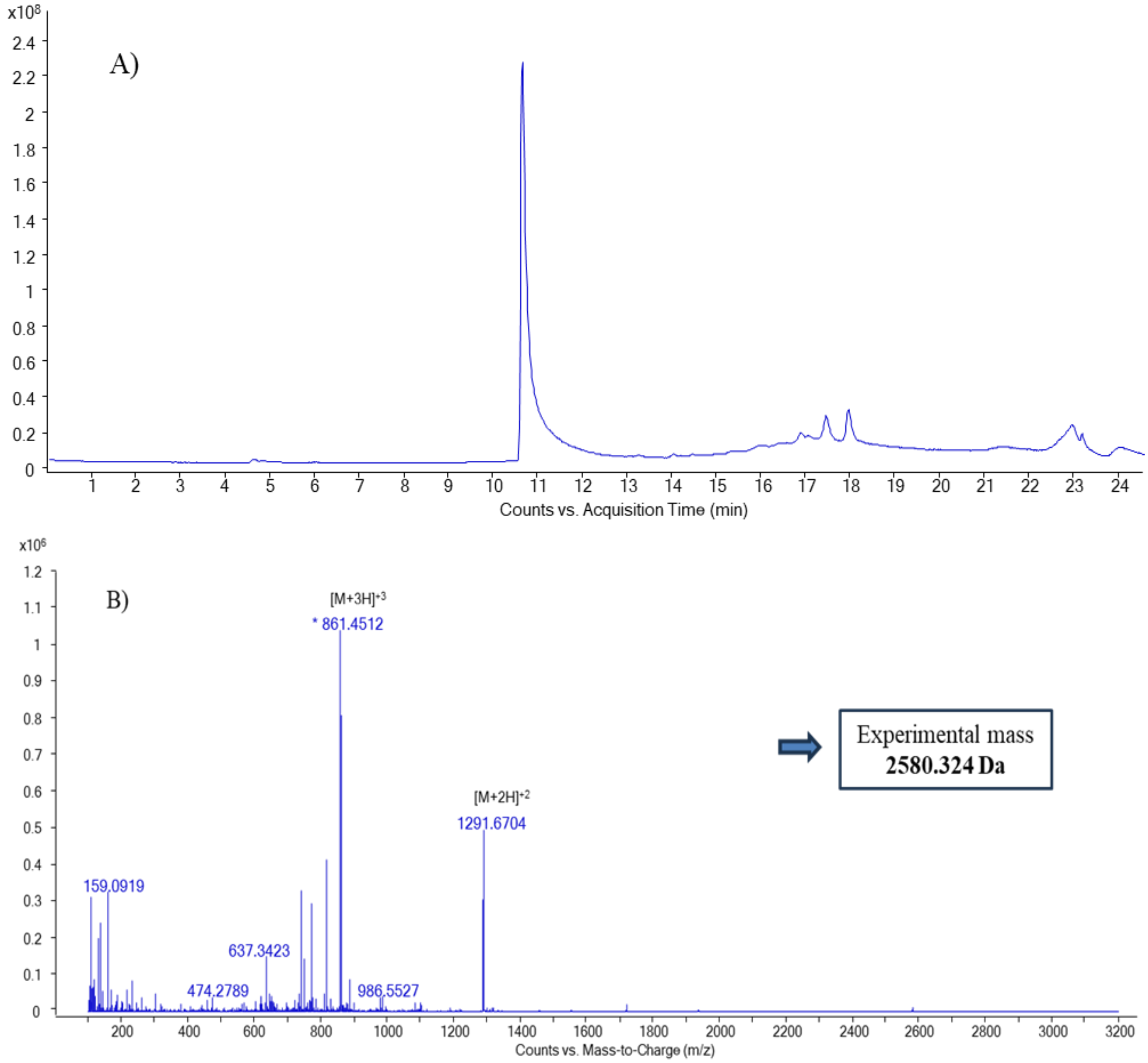

Figure S-4.- A) Extracted ion chromatogram of mMOG peptide (50 mg·L^{-1}) obtained by HPLC-MS. B) Mass spectrum of the peak eluting at 10.7 min.

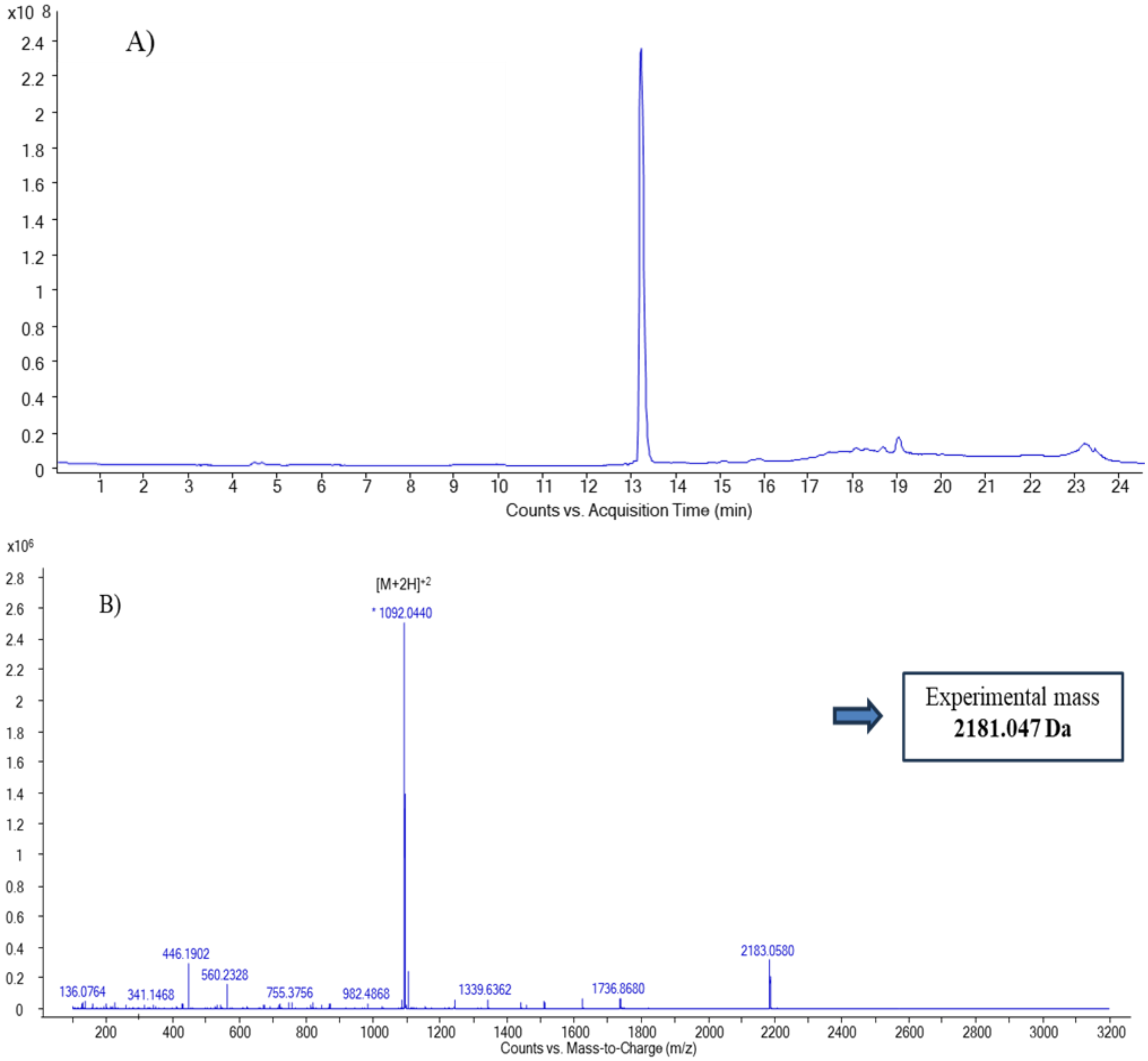

Figure S-5.- A) Extracted ion chromatogram of AChR peptide (50 mg·L^{-1}) obtained by HPLC-MS. B) Mass spectrum of the peak eluting at 13.3 min.

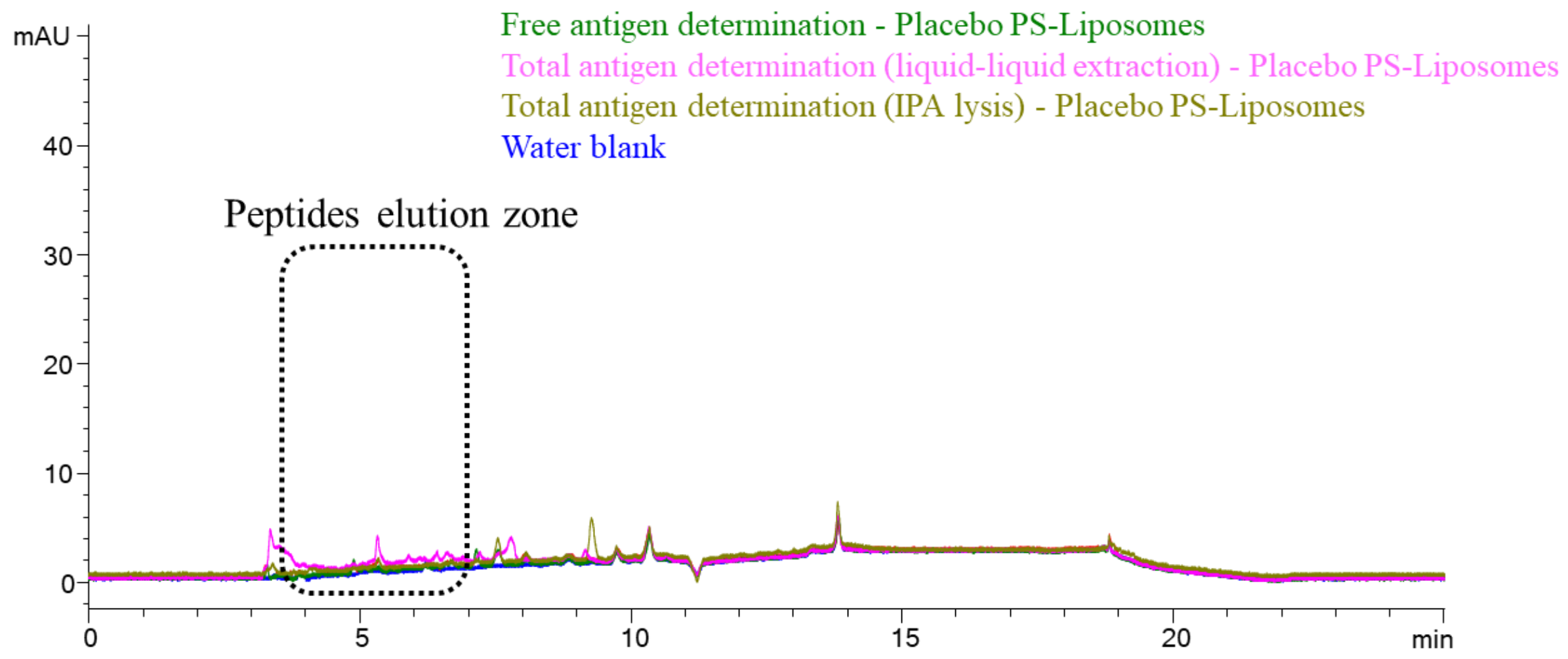

Figure S-6.- Comparison of the chromatograms obtained by HPLC-UV for the analysis of a water blank and a placebo PS-Liposome formulation after applying free (ultracentrifugation) and total (liquid-liquid extraction and 50% isopropanol (IPA) lysis) antigen determination protocols.

Table S-1.- Intra-day and inter-day precision and accuracy assessment for the analysis by HPLC-UV of each peptide solution at three concentration levels (20, 80 and 150 mg·L^{-1}).

**Peptide**	**Concentration (mg·L^{-1})**	**Intra-day (n=3)**		**Inter-day (n=3)**	
		Accuracy Recovery (%)	Precision RSD (%)	Accuracy Recovery (%)	Precision RSD (%)
**33-mer (CD)**	20	99.5	1.6	99.4	1.6
	80	99.2	0.2	99.1	0.5
	150	100.6	0.5	100.5	0.5
**Ins (T1D)**	20	100.6	0.7	100.2	0.8
	80	100.6	0.4	100.7	0.8
	150	99.9	1.2	100.2	1.2
**AChR (MG)**	20	101.2	0.4	99.7	2.9
	80	99.6	0.8	98.4	1.2
	150	99.9	0.4	100.4	0.5
**mMOG (MS)**	20	99.9	0.4	101.3	1.6
	80	100.5	0.8	100.6	0.7
	150	99.8	0.3	100.0	0.6

Table S-2.- Recoveries obtained when applying different methods for the determination of total peptide concentrations to spiked placebo PS-Liposomes with each antigenic peptide at 200 mg·L^{-1}.

Recoveries (%)	Spiked placebo PS-liposomes with peptide (200 mg·L^{-1})			
	**33-mer (CD)**	**Insulin (T1D)**	**AChR (MG)**	**mMOG (MS)**
**Liquid-liquid extraction**	98%	92%	72%	100%
**50% IPA**	109%	106%	89%	105%

Table S-3.- Fortified specificity recoveries obtained by HPLC-UV for placebo PS-Liposomes spiked with each peptide at three concentration levels (20, 80, and 150 mg·L^{-1}) after applying the selected total antigen determination protocol.

Spiked placebo PS-liposomes with peptide		
**Peptide**	**Concentration (mg·L^{-1})**	Recovery (%)
**33-mer (CD)**	20	101.4
	80	99.8
	150	99.7
**Ins (T1D)**	20	102.1
	80	100.6
	150	99.1
**AChR (MG)**	20	100.2
	80	100.5
	150	99.9
**mMOG (MS)**	20	101.0
	80	100.4
	150	99.6

Table S-4.- EE (%) of different PS-Liposome formulations applying the ultracentrifugation method for free antigen determination, and the selected method in each case for total antigen determination (liquid-liquid extraction for 33-mer, mMOG and AChR, and addition of 50% isopropanol for insulin)

	Batch 6<br>33-mer<br>(CD)	Batch 7<br>Insulin<br>(T1D)	Batch 8<br>AChR<br>(MG)	Batch 9<br>mMOG<br>(MS)
**EE (%)**	49%	76%	63%	85%
